# Food-Anticipatory Activity in Syrian Hamsters: Behavioral and Molecular Responses in the Hypothalamus According to Photoperiodic Conditions

**DOI:** 10.1371/journal.pone.0126519

**Published:** 2015-05-13

**Authors:** Rosana F. Dantas-Ferreira, Stéphanie Dumont, Sylviane Gourmelen, José Cipolla-Neto, Valérie Simonneaux, Paul Pévet, Etienne Challet

**Affiliations:** 1 Department of Neurobiology of Rhythms, Institute of Cellular and Integrative Neurosciences, UPR 3212 CNRS, University of Strasbourg, Strasbourg, France; 2 Department of Physiology and Biophysics, Institute of Biomedical Sciences, University of São Paulo, São Paulo, Brazil; Kent State University, UNITED STATES

## Abstract

When food availability is restricted, animals adjust their behavior according to the timing of food access. Most rodents, such as rats and mice, and a wide number of other animals express before timed food access a bout of activity, defined as food-anticipatory activity (FAA). One notable exception amongst rodents is the Syrian hamster, a photoperiodic species that is not prone to express FAA. The present study was designed to understand the reasons for the low FAA in that species. First, we used both wheel-running activity and general cage activity to assess locomotor behavior. Second, the possible effects of photoperiod was tested by challenging hamsters with restricted feeding under long (LP) or short (SP) photoperiods. Third, because daytime light may inhibit voluntary activity, hamsters were also exposed to successive steps of full and skeleton photoperiods (two 1-h light pulses simulating dawn and dusk). When hamsters were exposed to skeleton photoperiods, not full photoperiod, they expressed FAA in the wheel independently of daylength, indicating that FAA in the wheel is masked by daytime light under full photoperiods. During FAA under skeleton photoperiods, c-Fos expression was increased in the arcuate nuclei independently of the photoperiod, but differentially increased in the ventromedial and dorsomedial hypothalamic nuclei according to the photoperiod. FAA in general activity was hardly modulated by daytime light, but was reduced under SP. Together, these findings show that food-restricted Syrian hamsters are not prone to display FAA under common laboratory conditions, because of the presence of light during daytime that suppresses FAA expression in the wheel.

## Introduction

Physiological and behavioral processes are endogenously timed by the circadian system on a daily basis. The master clock located in the suprachiasmatic nucleus (SCN) of the hypothalamus is primarily synchronized by the daily variations of light intensity [1]. Besides the light–dark cycle, there are non-photic signals capable of entraining or modulating circadian rhythms, including cyclic food availability [2].

When restricted feeding schedules are imposed, animals manifest increased locomotor activity, called food-anticipatory activity (FAA), prior to food access. This behavioral response is best observed in nocturnal rodents when food is given during the light (resting) period, while their activity levels are normally low [3, 4]. FAA being expressed even in SCN-lesioned animals [5], a so-called feeding-entrainable clock or food clock, localized outside the SCN, has been proposed to be synchronized by meal time and to drive expression of FAA as a behavioral output. Many studies conducted to locate the food clock indicate that it may rely on a network of interacting brain structures. Among the candidates are hypothalamic nuclei, such as the dorsomedial nucleus (DMH), arcuate nucleus (ARC) and ventromedial nucleus (VMH) [6–8]. The metabolic hindbrain and the cerebellum are other possible components of the food clock network [9–11].

Most rodent species, such as rats and mice, and a wide number of other mammals are prone to display FAA when they are challenged with restricted feeding [4]. One notable exception is the Syrian Hamster [12] [13, 14]. In this study we tried to understand the reasons of the low meal anticipation expressed by this species. Several possibilities may explain why the Syrian hamster is not a standard meal anticipator. First, previous studies challenged hamsters with restricted feeding under long (i.e., summer-like) photoperiods [15]. As a photoperiodic species, Syrian hamsters display seasonal changes in adiposity, gonadal axis and circulating hormones, such as melatonin and sex steroids [16]. Hamsters may well be more sensitive to feeding cues under short (i.e., winter-like) photoperiods. Second, previous experiments analyzed behavioral responses of food-restricted hamsters using wheel-running activity [12, 14, 15, 17], a rewarding behavior that may influence food anticipation, as compared to general physical activity and food-bin approach [17]. Furthermore, wheel-running activity in hamsters can interfere with the photoperiodic integration [18]. Third, light during daytime has been recently shown to inhibit the expression of FAA in both nocturnal rats and mice [19]. Such a masking effect of light on FAA may be stronger in Syrian hamsters. To test these hypotheses, we conducted the present experiment in Syrian hamsters that were challenged with restricted feeding schedules under long (LP) or short (SP) photoperiods, with successive steps of full (Fu) and skeleton (Sk) photoperiods to unmask the putative inhibiting effects of daytime light on hamster FAA.

## Material and Methods

### Animals

Thirty Syrian hamsters *(Mesocricetus auratus*) bred in-house (Chronobiotron platform, UMS3415, CNRS and University of Strasbourg) were 4-month-old at the beginning of the experiments. From birth, they were maintained in LP consisting of 14-h light and 10-h dark (around 150 lux within the cages during the light period and dim red light at night), with lights on at 05:00, defining Zeitgeber time (ZT) 0, at 22 ± 1°C with *ad libitum* access to water and food, unless otherwise stated. The SP to which some animals were exposed 2 months prior to feeding schedules consisted of 10 h light and 14 h dark, with lights on at 07:00. This photoperiodic condition triggers an inhibition of the reproductive axis of the hamsters, leading to gonadal atrophy [16, 20]. All experiments were conducted in accordance with the French National Law (License 67–88) implementing the European Union Directive 2010/63/EU, and approved by the institutional animal research ethics board of the University of Strasbourg (#AL0212-11/06).

At the beginning of the experiments, hamsters were housed in individual cages with a running wheel. Animals had access to food via a 4 × 12-cm window cut through one end of the cage. Access to food during scheduled feeding was automatically controlled by electronic timers that permit the opening and closing of the food window using a metallic barrier (Intellibio, Seichamps, France). Under gaseous anesthesia [2% isoflurane in O_2_/N_2_O (50:50)], hamsters were i.p. implanted with E-mitter (battery free) telemetry devices (MiniMitter, Sunriver, OR, USA) that evaluate general locomotor activity. Wheel-running activity and general activity were recorded every 5 min (Vitalview acquisition system, MiniMitter). Food consumption and body weight were evaluated once a week.

### Lighting and feeding schedules

The effect of daytime light on behavior and physiology can be assessed by so-called skeleton (Sk) photoperiod. Experimental Sk usually consists in two daily 1-h light pulses mimicking dawn and dusk. Sk is considered as an experimental condition reflecting light sampling behavior of nocturnal rodents in their natural environment [21]. This appears especially relevant for species, such as Syrian hamsters, that rest in a burrow in the wild and that do not receive light cues during most of the light phase. Sk is known to maintain photic entrainment of the master clock in nocturnal rodents [22, 23]. When rodents are fed *ad libitum*, Sk conditions do not affect the daily rhythms of motor activity, feeding and drinking [22, 23]. Of particular interest for the present study, Sk has recently been shown to enhance (i.e., unmask) the expression of FAA in nocturnal rats and mice fed during daytime [19]. Furthermore, timing changes between the two light pulses trigger photoperiodic responses in the Syrian hamster, as do changes in full photoperiods [24].

**Fig 1:**
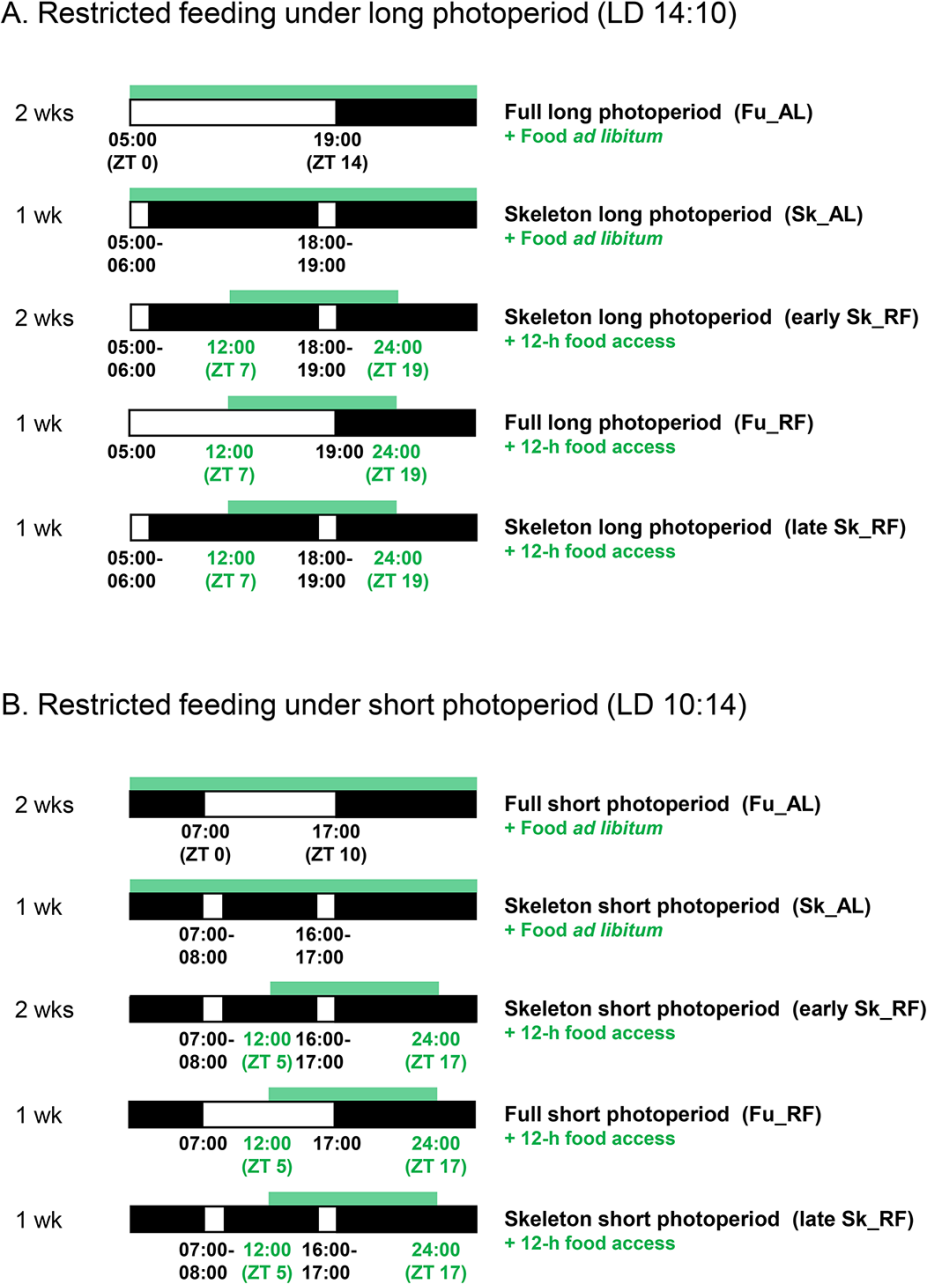
Timetable of photoperiodic and feeding conditions. Panel A: Restricted feeding under long photoperiod. Black and white bars indicate daily periods of darkness and light, respectively. Zeitgeber time (ZT) 0 and ZT 14 correspond to lights on and lights off, respectively. Green shaded areas show the daily 12-h period of food access during food restriction, from midday (i.e., ZT 7) to ZT 19. On the left of each horizontal bar (corresponding to a given photoperiodic and feeding condition) is shown the duration of that step in weeks (wk). Fu, full long long photoperiod; Sk, skeleton long photoperiod (comprising 1-h light from ZT 0 to ZT 1 followed by 12-h dark, and then 1-h light from ZT 13 to ZT 14, and 10-h dark); RF, restricted feeding (daily food access during 12 h, from ZT 7 to Z 19); early Sk_RF, first period of restricted feeding under skeleton long photoperiod; late Sk_RF, second period of restricted feeding under skeleton long photoperiod. Panel B: Restricted feeding under short photoperiod. Black and white bars indicate daily periods of darkness and light, respectively. Zeitgeber time (ZT) 0 and ZT 10 correspond to lights on and lights off, respectively. Green shaded areas show the daily 12-h period of food access during food restriction, from midday (i.e., ZT 5) to ZT 17. On the left of each horizontal bar (corresponding to a given photoperiodic and feeding condition) is shown the duration of that step in weeks (wk). Fu, full short photoperiod; Sk, skeleton short photoperiod (comprising 1-h light from ZT 0 to ZT 1 followed by 8-h dark, and then 1-h light from ZT 9 to ZT 10, and 14-h dark); RF, restricted feeding (daily food access during 12 h, from ZT 5 to Z 17); early Sk_RF, first period of restricted feeding under skeleton short photoperiod; late Sk_RF, second period of restricted feeding under skeleton short photoperiod.

A first group of 15 animals were exposed to the following conditions of LP:

After a baseline period (2 wks) to Fu_LP, free-fed animals were exposed for 3 wks to a 1^st^ period (i.e., early) of Sk that consisted of 1-h light (150 lux) from ZT 0 to ZT 1, 12-h dark from ZT 2 to ZT 13 then 1-h light from ZT 13 to ZT 14, followed by 10-h dark from ZT 14 to ZT 24. The 1^st^ wk under Sk with food *ad libitum* (Sk_AL) was followed by 4 wks of food restriction for 9 hamsters, while 6 control animals were kept with food *ad libitum*. The restricted animals had access to food for 12 h starting in the middle of the light period (from ZT 7 to ZT 19). After 2 wks of restricted feeding under early Sk (early Sk_RF), animals were transferred for 1 wk to Fu, while restricted feeding was maintained (Fu_RF). Thereafter, RF hamsters were exposed for a 2^nd^ period of 1 wk to Sk (i.e., late Sk_RF) (Fig 1A).

A second group of 15 animals were exposed to the following conditions of SP:

Following 2 wks of Fu_SP, hamsters fed *ad libitum* were transferred to a 1^st^ period (i.e., early) Sk that consisted of 1-h light (150 lux) from ZT0 to ZT 1, then 8-h dark from ZT 1 to ZT 9 and 1-h light from ZT9 to ZT 10, followed by 14-h dark from ZT 10 to ZT 24. One wk later, 9 hamsters were challenged with restricted feeding, daily access to food being limited to 12 h starting in the middle of the light period (from ZT 5 to ZT 17), while 6 control animals were kept with food *ad libitum*. After 2 wks of restricted feeding under early Sk (early Sk_RF), food-restricted animals were transferred for 1 wk to Fu (Fu_RF). Then, hamsters were exposed for a 2^nd^ period of 1 wk to Sk (i.e., late Sk_RF) (Fig 1B).

At the end of the experiment, all animals were euthanized with an overdose of pentobarbital (200 μL/100 g body mass; solution at 54.7 mg/ml; CEVA, La Ballastiere, France) during the middle of the light period (ZT 6–7 and ZT 4–5 for LP and SP, respectively), corresponding to the hour prior to food access in food-restricted animals. Blood was collected by intra-cardiac puncture and transferred in tubes containing EDTA 4%. The animals were then perfused with 0.9% saline followed by 4% paraformaldehyde in 0.1 PBS. The brains were removed, post-fixed in the same fixative overnight, cryoprotected in 30% sucrose, frozen in pre-cooled isopentane and stored at -80°C. Serial 30-μm coronal sections of the brains obtained with a cryostat (CM3050, Leica Biosystems, Nanterre, France) were immersed in a cryoprotectant solution and stored in -20°C until assay.

### Actimetry recordings and analysis

Wheel rotations were recorded using VitalView Software (MiniMitter Co., Sunriver, OR, USA). The analysis of the data was performed using Matlab 7.0 device and Clocklab data acquisition interface and software (Actimetrics, Evanston, IL, USA). The average amount of activity was calculated for the 7 last days of each of the 5 conditions in both photoperiods as described above. The parameters analyzed were Total Wheel-running Activity and Total General Activity. FAA in food-restricted hamsters and corresponding activity in control animals (thereafter called Midday Wheel-running Activity and Midday General Activity) were defined as locomotor activity performed during the 2 h previous to food access (i.e., from ZT 3 to ZT 5 and from ZT 5 to ZT 7 in SP and LP, respectively).

### Cortisol assay

Plasma concentrations of cortisol were determined by a Cortisol Express EIA Kit (AYN830, Cayman Chemical, Ann Arbor, MI, USA). The limit of sensitivity of the assay was 0.1 ng·mL^−1^.

### c-Fos immunostaining

For c-Fos immunostaining, the hypothalamic sections were pre-treated for 30 minutes in PBS solution with 3% H_2_O_2_ and then treated with PBS blocking solution containing 10% goat serum for 2 h in cold room. The sections were then incubated in solution containing the first antibody anti c-Fos made in rabbit (sc-52, Santa Cruz Biotechnology, Santa Cruz, CA, USA) in a concentration of 1:10.000 and 10% goat serum for 24 h. Then the sections were incubated with biotinylated second antibody anti rabbit made in goat (Vector Laboratories, Burlingame, CA, USA) in a concentration of 1:500 for 2 h in cold room. Then they were incubated with Vectastain ABC Kit (Vector Laboratories) in a concentration of 1:250 for 1 hour and then colored signal was obtained using 3,3 Diaminobenzidine Tetrahydrochloride (0.5 mg/ml; Sigma Aldrich, St Louis, MO, USA) and H_2_O_2_ (0.015%) to activate the chromogen. Sections were mounted in glass slides and let dry overnight, dehydrated and coverslipped. Brightfield images were obtained with a microscope (DMRB, Leica) using 10X objective lens. The images were obtained using a digital camera (DP50, Olympus Imaging, Center Valley, PA, USA).

### Neuroanatomical quantitative analyses

The anatomical regions were defined based on “A Stereotaxic Atlas of the Golden Hamster Brain” [25]. C-Fos immunoreactive cells were bilaterally counted in sections corresponding to the rostro-caudal level -0.6 mm from bregma for SCN, and to the levels -2 mm from bregma for VMH and DMH and -2.4 mm for ARC.

To quantify c-Fos immunoreactive cells in each brain structure we used ImageJ software (NIH, Besthesda, USA). Data were expressed as number of immunoreactive cells per region.

### Statistical analysis

Results are shown as means ± SEM. Data were analyzed by two-way ANOVA (photoperiod x feeding conditions) or three-way ANOVA (mixed linear model: photoperiod x feeding x baseline vs. final step) with repeated measures, followed by Bonferroni post-hoc test. Statistical significance was set at p<0.05.

## Results

### Changes in body mass and food intake according to photoperiod and feeding schedules

Despite the fact that food access was maintained for 12 h/day, food-restricted hamsters lost body mass. Body mass loss, however, did not differ according to the photoperiod (23 and 24%, in SD and LD, respectively; Table 1). Of note, such a body mass loss occurred in spite of essentially unchanged food intake, whatever the photoperiod (Table 1, including statistical results).

**Table 1:** Changes of body mass and food intake in food-restricted Syrian hamsters exposed to skeleton short or long photoperiod.

Step	Feeding group	Body mass (g)	Food intake (g/day)
Baseline SP	Control fed	122.2 ± 4.8	7.8 ± 0.5
End SP	Control fed	124.1 ± 5.0	7.4 ± 0.4
Baseline SP	Food-restricted	125.4 ± 3.7^a^	8.6 ± 0.6
End SP	Food-restricted	96.6 ± 5.3^b^	6.7 ± 0.2
Baseline LP	Control fed	112.1 ± 4.8	6.4 ± 0.4
End LP	Control fed	112.6 ± 3.8	7.0 ± 0.4
Baseline LP	Food-restricted	124.3 ± 3.3^a^	6.9 ± 0.5
End LP	Food-restricted	94.7 ± 3.2^b^	6.5 ± 0.3

Control hamsters fed *ad libitum* (n = 6) and food-restricted hamsters (n = 9); SP: skeleton short photoperiod; LP: skeleton long photoperiod. Data were analyzed with a mixed linear model (3-way ANOVA with repeated measures). For body mass, main effect of photoperiod: F_(1,26)_ = 2.61, p = 0.12); main effect of feeding condition: F_(1,26)_ = 3.98, p = 0.057; main effect of step: F_(1,28)_ = 41.95, p<0.001; [feeding x step] interaction: F_(1,28)_ = 49.67, p<0.001. For food intake, main effect of feeding condition: F_(1,27)_ = 2.17, p = 0.15; main effect of step: F_(1,29)_ = 2.37, p = 0.13; main effect of photoperiod: F_(1,27)_ = 0.62, p = 0.44 and none interaction was significant. In a given column, groups with a different superscript (a or b) were significantly different.

### Photoperiod and light schedule changes in locomotor activity

**Fig 2:**
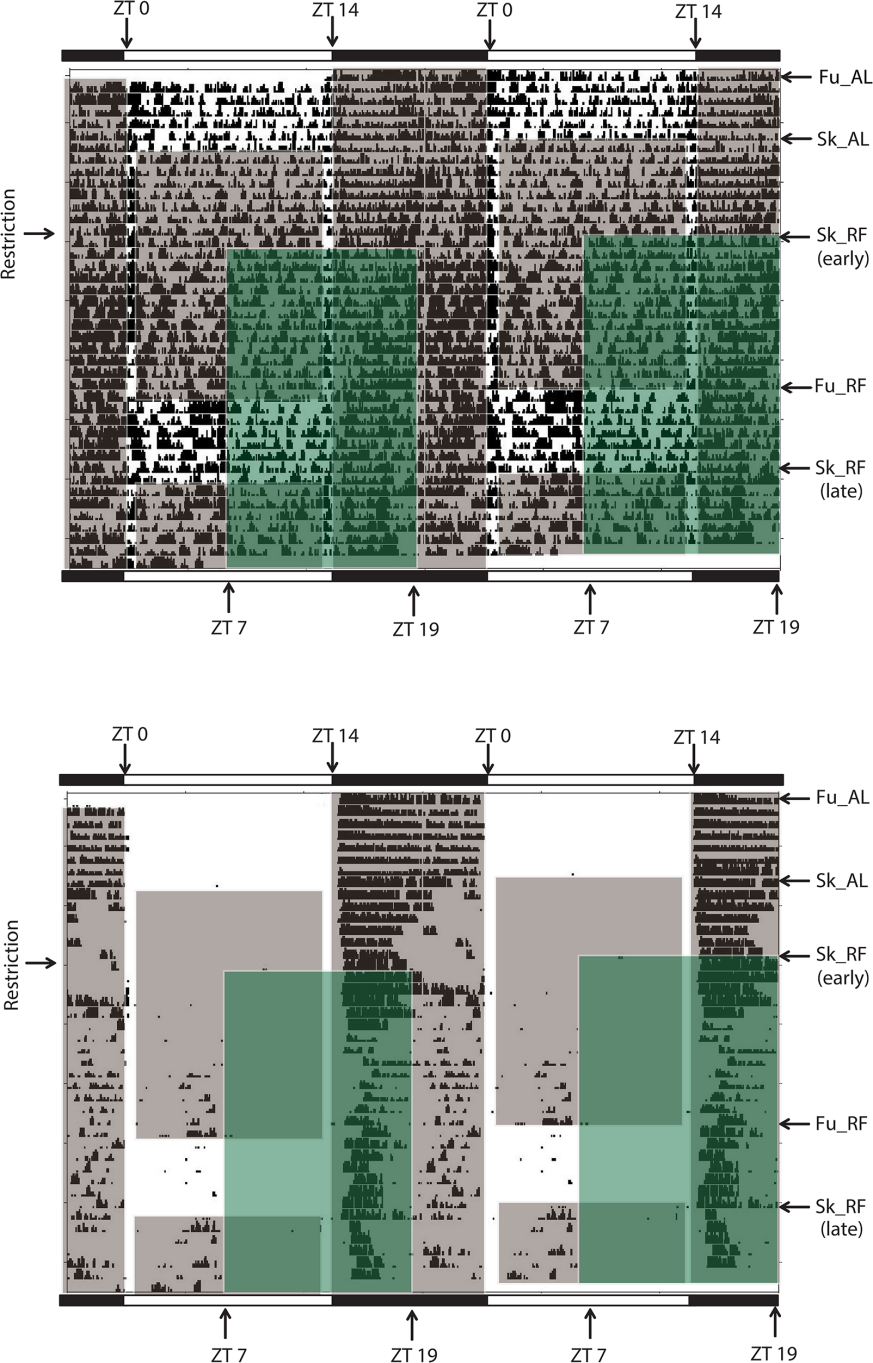
Food-anticipatory activity in a hamster exposed to a long photoperiod. Panel A: wheel-running activity; panel B: general cage activity. Each line represents 48 h, plotted in 5-min bins. The initial light-dark cycle (14 h of light, 10 h of darkness; full long photoperiod) is indicated by horizontal white and black bars. Zeitgeber time (ZT) 0 corresponds to lights on. Gray shaded areas indicate periods of darkness. Arrows on the right Y axis show the different photoperiodic conditions (Sk: skeleton long photoperiod, comprising 1-h light from ZT 0 to ZT 1 followed by 12-h dark, and then 1-h light from ZT 13 to ZT 14, and 10-h dark; Fu: full long photoperiod, comprising 14 h of light and 10 h of dark). During baseline, hamsters were fed *ad libitum* (AL). Green shaded areas show the daily 12-h period of food access during restricted feeding (RF), from midday (i.e., ZT 7) to ZT 19. Arrows on the left Y axis show the onset of food restriction. Arrows next to Sk_RF (early) and Sk_RF (late) on the right Y axis show the first and second periods of restricted feeding under Sk, respectively.

**Fig 3:**
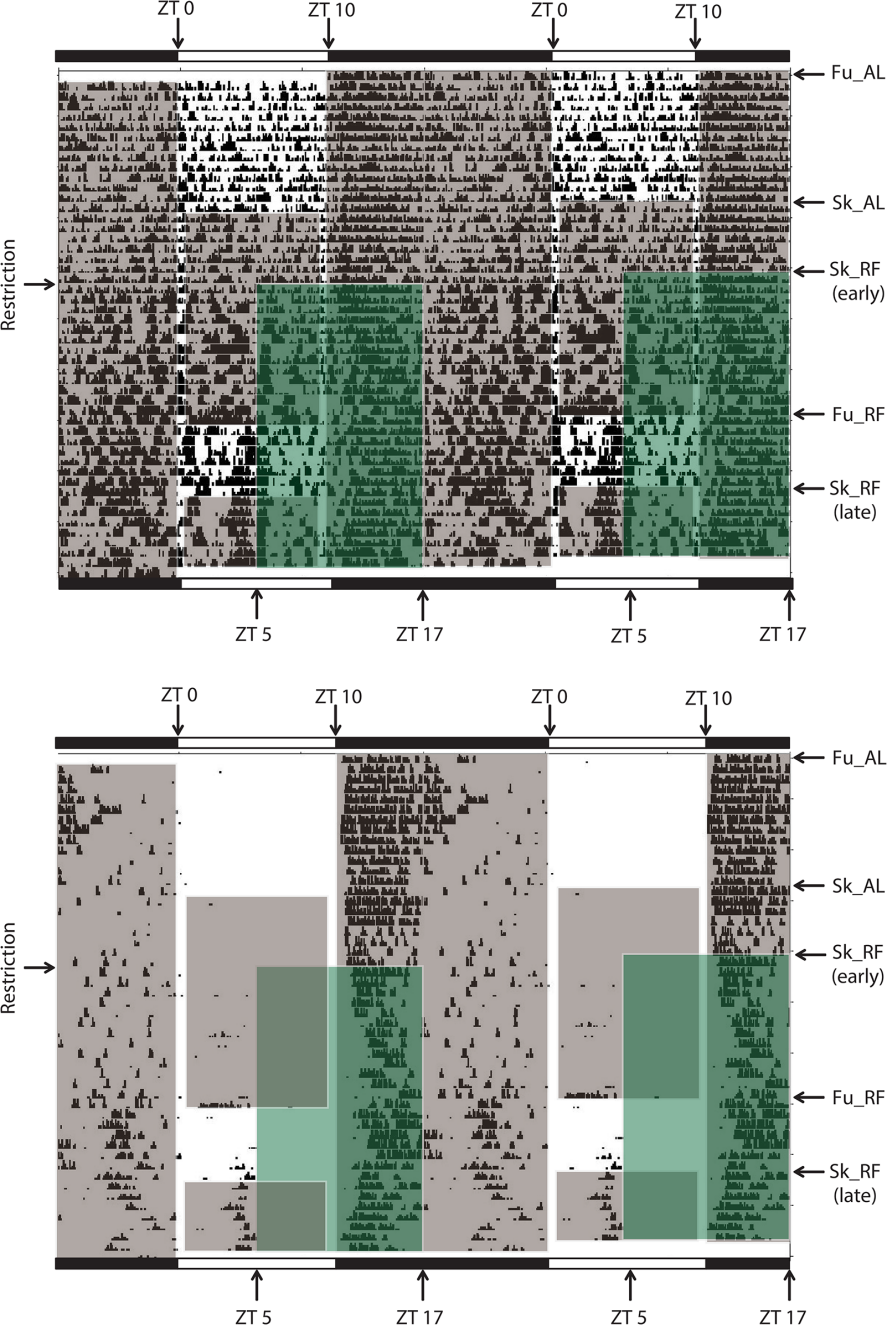
Food-anticipatory activity in a hamster exposed to a short photoperiod. Panel A: wheel-running activity; panel B: general cage activity. Each line represents 48 h, plotted in 5-min bins. The initial light-dark cycle (10 h of light, 14 h of darkness; full short photoperiod) is indicated by horizontal white and black bars. Zeitgeber time (ZT) 0 corresponds to lights on. Gray shaded areas indicate periods of darkness. Arrows on the right Y axis show the different photoperiodic conditions (Sk: skeleton long photoperiod, comprising 1-h light from ZT 0 to ZT 1 followed by 12-h dark, and then 1-h light from ZT 9 to ZT 10, and 14-h dark; Fu: full short photoperiod, comprising 10 h of light and 14 h of dark). During baseline, hamsters were fed *ad libitum* (AL). Green shaded areas show the daily 12-h period of food access during restricted feeding (RF), from midday (i.e., ZT 5) to ZT 17. Arrows on the left Y axis show the onset of food restriction. Arrows next to Sk_RF (early) and Sk_RF (late) on the right Y axis show the first and second periods of restricted feeding under Sk, respectively.

Locomotor activity was assessed by both wheel-running activity and general cage activity (Figs 2 and 3).

**Fig 4:**
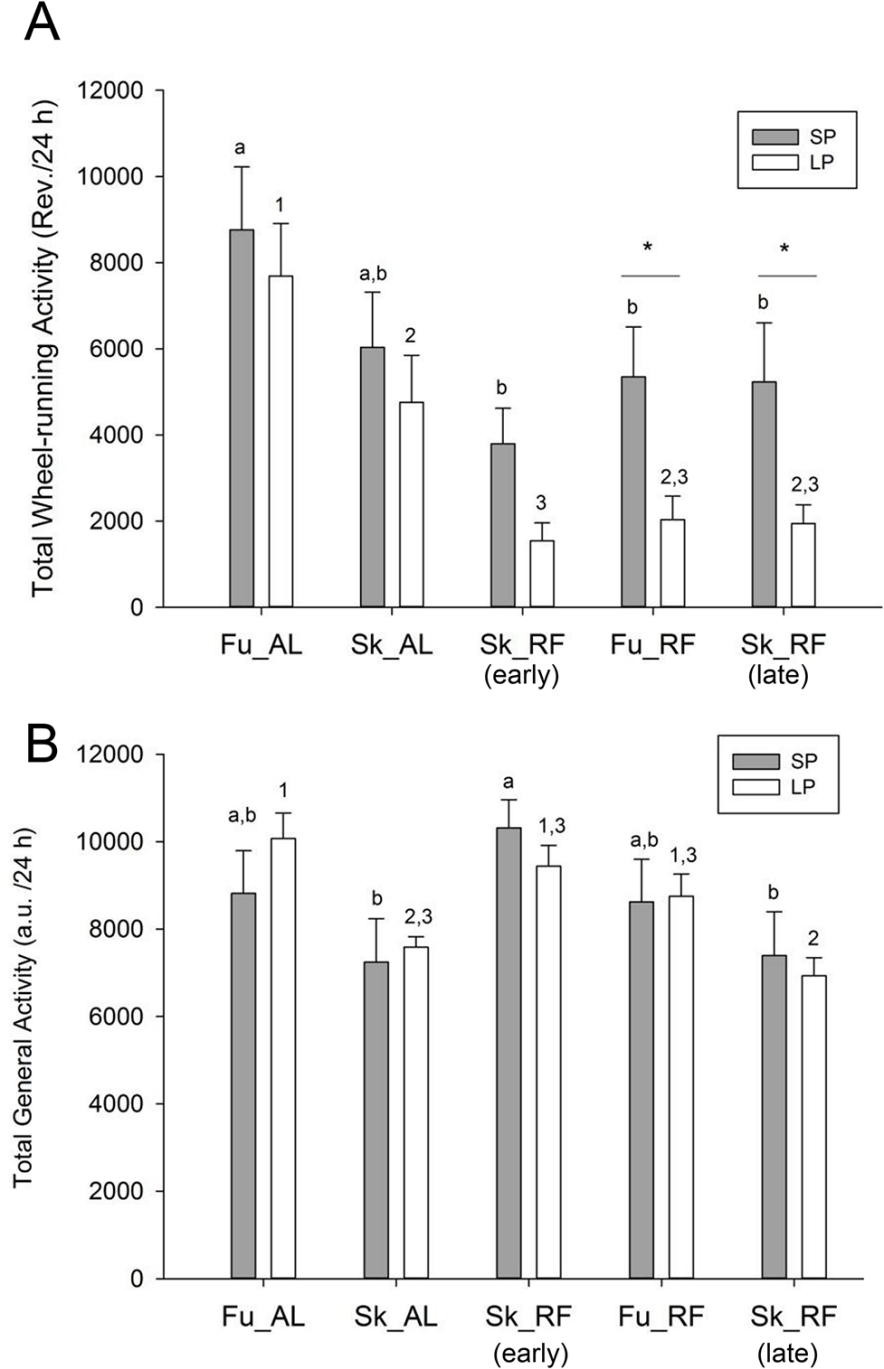
Total activity in hamsters exposed to long or short photoperiods. This figure shows the daily level of wheel-running activity (panel A) and general cage activity (panel B). Data for short and long photoperiod are presented with grey and white histograms, respectively. Within data for short photoperiod, significant differences (*p<*0.05) are shown by means lacking common letters. Within data for long photoperiod, significant differences (*p<*0.05) are indicated by means lacking common numbers. For a given step between long and short photoperiods, pairwise differences (*p<*0.05) are shown with stars. a.u., arbitrary units; Rev., wheel revolutions; Fu, full photoperiod; Sk, skeleton photoperiod; AL, food *ad libitum*; RF, restricted feeding (12-h food access); Early and late Sk_RF, first and second periods of restricted feeding under Sk, respectively.

Regarding to total wheel-running activity, there was a trend for an effect of photoperiod (F_(1,64)_ = 3.59; p = 0.076); a significant effect of feeding (F_(4,64)_ = 19.12, p<0.001), while [photoperiod x feeding] interaction was not significant (F_(4, 64)_ = 1.12, p = 0.35) (Figs 2, 3 and 4A). When food was provided *ad libitum*, there was no difference according to the photoperiodic regimen, except that it was reduced by Sk under LP. When restricted feeding was imposed, there was a reduction of total activity in both photoperiods, with more marked effects under LP.

**Fig 5:**
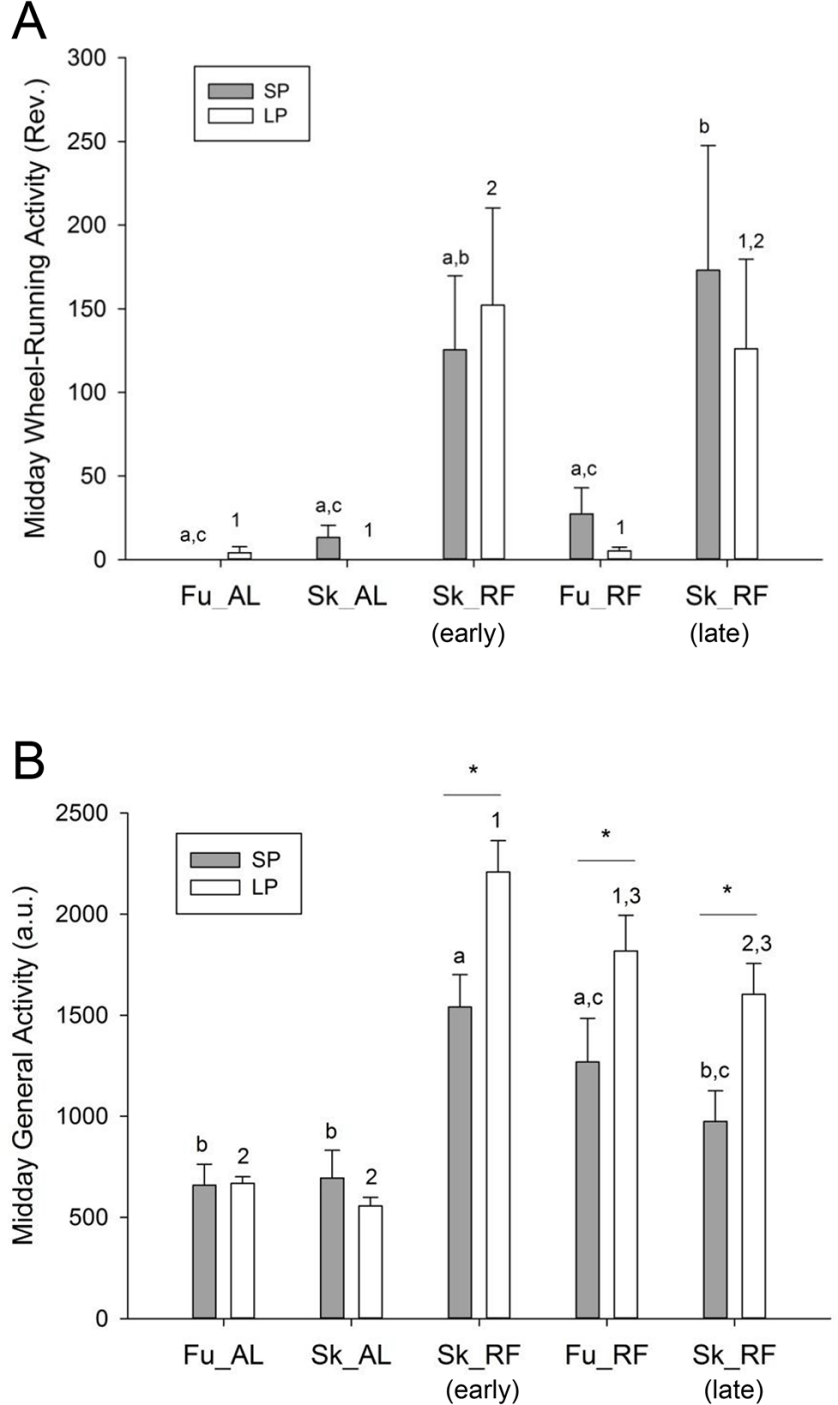
Midday activity in hamsters exposed to long or short photoperiods. This figure shows the level of wheel-running activity (panel A) and general cage activity (panel B) during the 2 h before midday period (i.e., from ZT 3 to ZT5, and form ZT 5 and ZT 7 in short and long photoperiods, respectively). During restricted feeding (RF), this corresponds to the period before food access, during which animals may express food-anticipatory activity. Data for short and long photoperiod are shown by grey and white histograms, respectively. Within data for short photoperiod, significant differences (*p<*0.05) are shown by means lacking common letters. Within data for long photoperiod, significant differences (*p<*0.05) are indicated by means lacking common numbers. For a given step between long and short photoperiods, pairwise differences (*p<*0.05) are shown with stars. a.u., arbitrary units; Rev., wheel revolutions; Fu, full photoperiod; Sk, skeleton photoperiod; AL, food *ad libitum*; RF, restricted feeding (12-h food access). Early and late Sk_RF, first and second periods of restricted feeding under Sk, respectively.

FAA in the wheel was modified by feeding conditions, but not by photoperiod (Main effect of photoperiod: F_(1,64)_ = 0.12; p = 0.73; Main effect of feeding: F_(4,64)_ = 9.22, p<0.001; [photoperiod x feeding] interaction: F_(4, 64)_ = 0.32, p = 0.86). In animals fed *ad libitum*, wheel-running activity in late morning was low under both Fu and Sk conditions for both photoperiods. A higher midday activity (FAA) was observed during food restriction as compared to baseline conditions of food *ad libitum*. This bout of activity was masked by daytime light, since food-restricted animals exposed to the full LP or SP reduced their wheel-running activity to levels found in *ad libitum* fed animals (Fig 5A). Furthermore, the level of FAA in the wheel increased again under late Sk, to the same extent as in early Sk for both photoperiodic conditions (Fig 5).

Total general activity was modified by feeding conditions, but not by photoperiod (Main effect of photoperiod: F_(1,64)_ = 0.009; p = 0.92; Main effect of feeding: F_(4,64)_ = 10.79, p<0.001; [photoperiod x feeding] interaction: F_(4, 64)_ = 1.24, p = 0.30) (Figs 2 and 4B). Under Sk SP, total general cage was initially increased by restricted feeding (i.e., early Sk_RF), as compared to food *ad libitum*. This effect was no longer significant in late Sk_RF. In LP, there was a reduction of total general activity in ad lib fed hamsters transferred to Sk, an effect that was counteracted by RF. Late Sk_RF was also associated with a reduction in total level of general activity, in comparison to early Sk_RF.

FAA measured with general cage activity was affected by photoperiod F_(1,64)_ = 6.12; p = 0.025, feeding conditions F_(4,64)_ = 43.63, p<0.001), and [photoperiod x feeding] interaction was also significant (F_(4,64)_ = 5.20, p = 0.001). Level of midday general activity was low when hamsters were fed *ad libitum* in both SP and LP. FAA of general activity during the 2 h before food availability was higher under LP than in SP. Exposure to early Sk or Fu did not markedly modify FAA of general activity, irrespective of photoperiod. The level of FAA measured by general activity under late Sk, however, was found to be decreased as compared to early Sk, but not Fu, for both photoperiods (Fig 5B).

**Fig 6:**
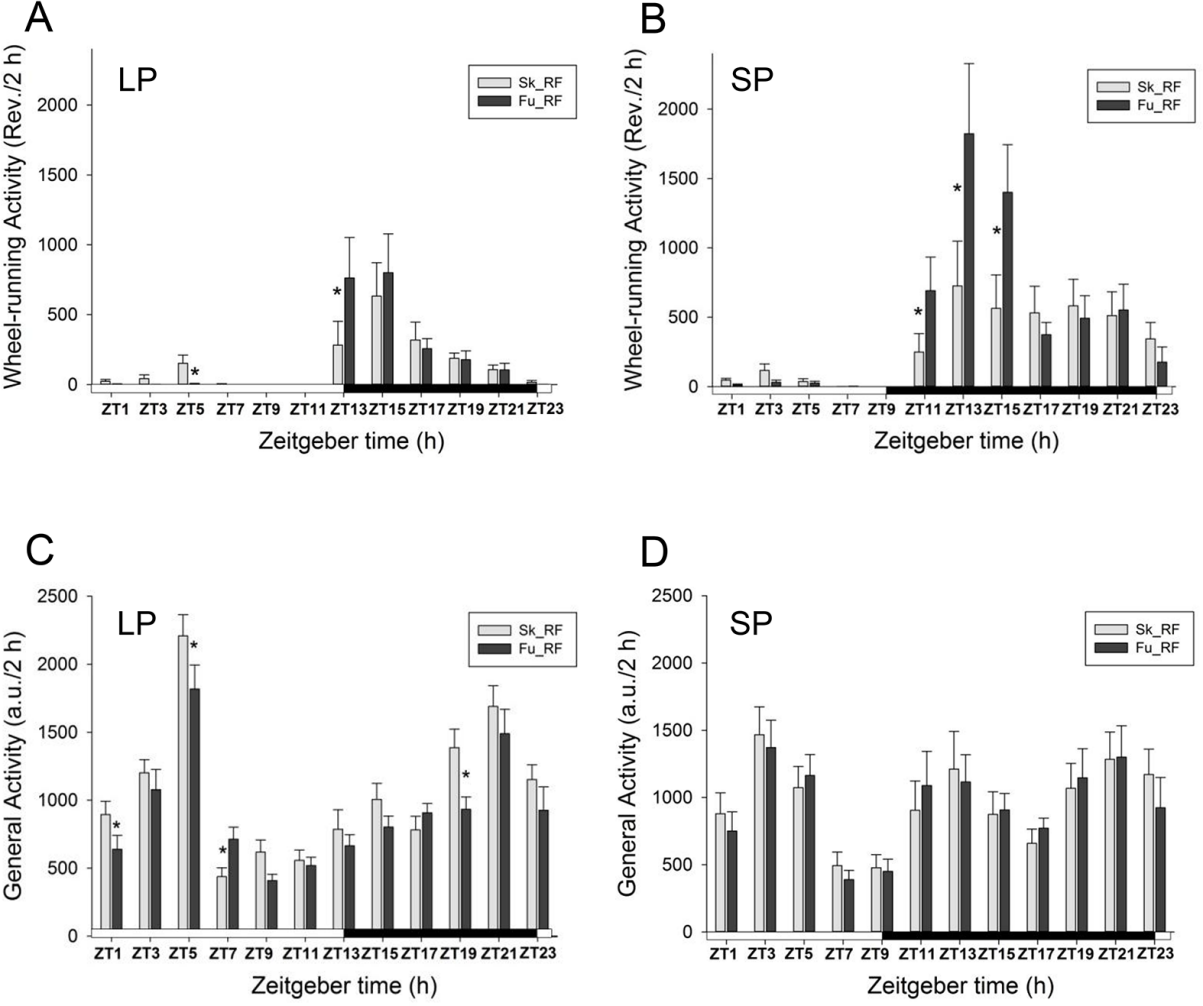
Activity profiles in food-restricted hamsters exposed to skeleton or full long and short photoperiods. This figure illustrates the daily profile of wheel-running activity (upper panels: A and B) and general cage activity (lower panels: C and D) over 24 h in food-restricted hamsters exposed to early skeleton (Sk_RF; light grey histograms) and full photoperiods (Fu_RF; dark grey histograms) under long (LP, left panels: A and C) and short photoperiods (SP, right panels: B and D). Data are presented as 2-h intervals per 24 h (ZT captions below X axis indicate the beginning of each of the 12 2-h intervals). Data for ZT5-7 are replotted from Fig 5. Horizontal white and black bars represent the full photoperiods (14 h of light and 10 h of darkness for full long photoperiod in panels A and C; 10 h of light and 14 h of darkness for full short photoperiod in panels B and D). For a given ZT, pairwise differences between Sk_RF and Fu_RF (*p<*0.05) are shown with stars. a.u., arbitrary units; Rev., wheel revolutions; Fu, full photoperiod; Sk, skeleton photoperiod; RF, restricted feeding (12-h food access); ZT, Zeitgeber time.

Next, we analyzed in more detail the daily profiles of early Sk_RF and Fu_RF in both photoperiods. The level of wheel-running activity was affected by ZT in LP and SP (F_(11,88)_ = 5.8, p<0.001 and F_(11,88)_ = 7.5, p<0.001, respectively). Moreover, the [photoperiod x feeding] interaction was significant for both photoperiods (F_(11,88)_ = 4.7; p<0.001 and F_(11,88)_ = 5.0, p<0.001, respectively). In LP, there was a sharp (30-fold) decrease of wheel-running at ZT5 under Fu_RF compared to early Sk_RF (Fig 6A), reflecting the masking of light on FAA already reported above (Fig 5A). In addition, wheel-running activity during RF was markedly increased (2.5-fold) in early night under Fu_LP (ZT13) and Fu_SP (at ZT11, ZT13 and ZT15) as compared to respective early Sk conditions (Fig 6A and 6B). The level of general cage activity was also changed according to ZT in LP and SP (F_(11,88)_ = 23.8, p<0.001 and F_(11,88)_ = 4.6, p<0.001, respectively). Moreover, the [photoperiod x feeding] interaction was significant, but only under LP photoperiod (F_(11,88)_ = 2.7; p<0.01 in LP and F_(11,88)_ = 1.2, p = 0.3 in SP). More specifically under LP, there were modest decreases in general activity at ZT1, ZT5 and ZT19, as well as an increase at ZT7 in Fu_RF in comparison to early Sk_RF (Fig 6C). No changes in general activity according to lighting conditions (i.e., between early Sk_RF and Fu_RF) were detected in SP (Fig 6D).

### Photoperiodic and feeding schedules changes in plasma cortisol levels

Plasma cortisol was affected neither by photoperiod (F_(1,24)_ = 0.69; p = 0.41), nor by feeding conditions (F_(1,24)_ = 0.02; p = 0.88), indicating that it did not increase during FAA; Table 2). Previous studies have found increased levels of morning cortisol in food-restricted Syrian hamsters [26]. Thus, the lack of significant increased cortisol in the morning might be due to inappropriate timing. Further studies are warranted to investigate the daily profiles of cortisol in food-restricted hamsters.

**Table 2:** Plasma cortisol in food-restricted Syrian hamsters exposed to short or long skeleton photoperiod.

Step	Feeding group	Plasma cortisol (ng/mL)
End SP	Control fed	18.9 ± 3.9
End SP	Food-restricted	20.1 ± 2.4
End LP	Control fed	16.9 ± 4.7
End LP	Food-restricted	16.7 ± 1.8

Control hamsters fed *ad libitum* (n = 6) and food-restricted hamsters (n = 9); SP: skeleton short photoperiod; LP: skeleton long photoperiod. Data were analyzed with 2-way ANOVA.

### Photoperiodic and feeding schedules changes in c-Fos expression

In the ARC, neuronal activity evaluated by c-Fos expression was always higher in food-restricted hamsters as compared to *ad libitum* fed controls, independently of the photoperiodic conditions (main effect of feeding: F_(1,20)_ = 16.04; p<0.001; main effect of photoperiod: F_(1,20)_ = 2.74; p = 0.11; [photoperiod x feeding] interaction: F_(1,20)_ = 0.43; p = 0.52).

In the SCN, no difference in c-Fos expression was detected according to the photoperiodic regimen (main effect of photoperiod: F_(1,20)_ = 0.04; p = 0.85) or the feeding schedule (main effect of feeding: F_(1,20)_ = 0.11; p = 0.74; [photoperiod x feeding] interaction: F_(1,20)_ = 1.12; p = 0.30).

In the DMH, the pattern of c-Fos activation was close to the one observed in ARC, with more marked effects under Sk SP (main effect of feeding: F_(1,20)_ = 11.25; p = 0.003; main effect of photoperiod: F_(1,20)_ = 4.05; p = 0.06; [photoperiod x feeding] interaction: F_(1,20)_ = 0.34; p = 0.57).

**Fig 7:**
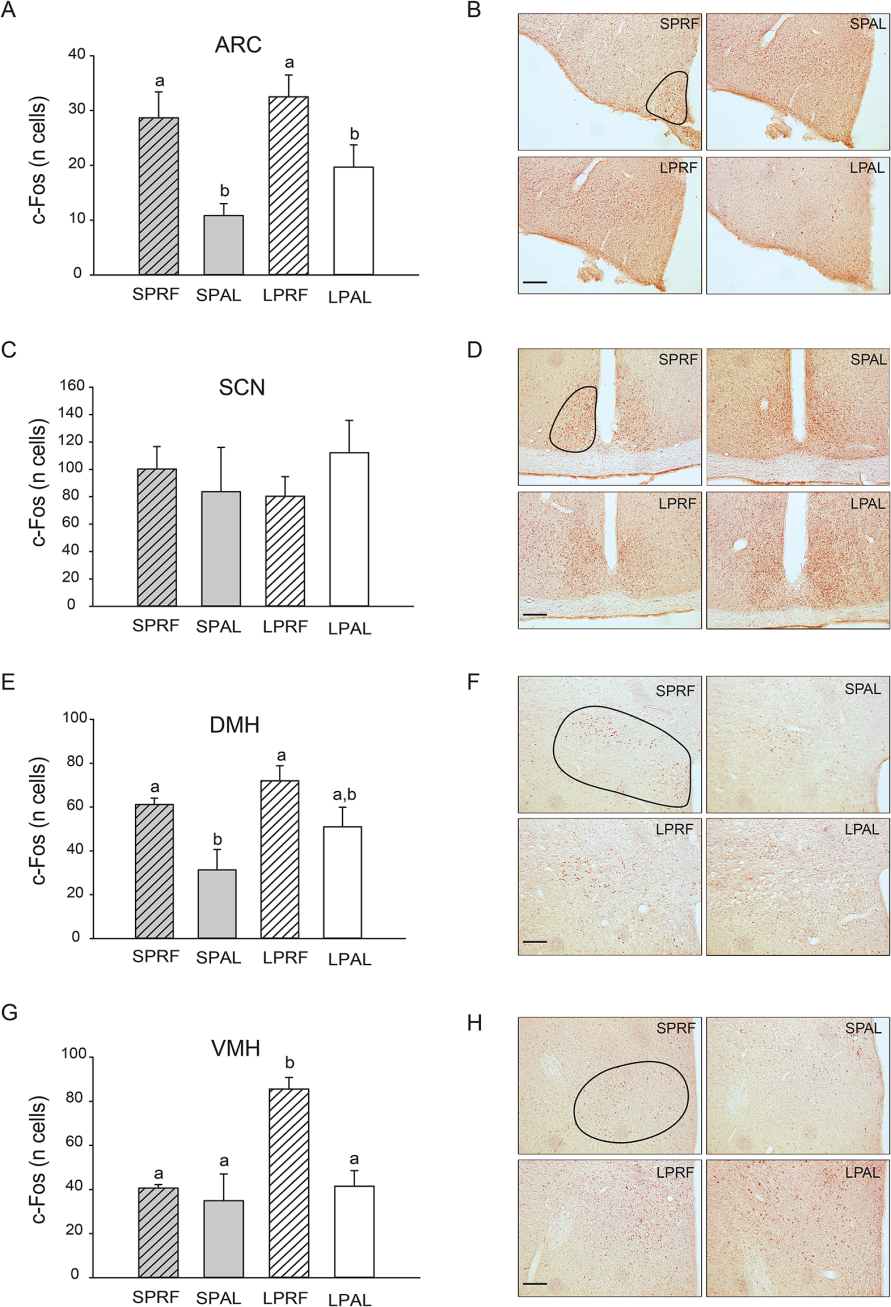
c-Fos responses in the hypothalamus of food-restricted hamsters exposed to skeleton long and short photoperiods. c-Fos mean responses in the arcuate (ARC, panel A), suprachiasmatic (SCN, panel C), dorsomedial (DMH, panel E), and ventromedial nuclei (VMH, panel G) of Syrian hamsters fed *ad libitum* or challenged with timed 12-h food access under long (LP) or short photoperiods (SP). Groups with different letters are significantly different (*p*<0.05). Representative photomicrographs of c-Fos staining in left ARC (panel B), bilateral SCN (panel D), left dorsomedial (panel F), and left VMH (panel H). For each level, the lined shape shows its location. Scale bar: 150 μm.

In the VMH, a higher expression of c-Fos was found only in food-restricted animals exposed to Sk LP (main effect of feeding: F_(1,20)_ = 11.60; p = 0.003; main effect of photoperiod: F_(1,20)_ = 10.86; p = 0.004; [photoperiod x feeding] interaction: F_(1,20)_ = 6.37; p = 0.02) (Fig 7).

## Discussion

This study demonstrates that food anticipation is expressed by Syrian hamsters exposed to skeleton photoperiods, indicating that FAA in the wheel is notably diminished (i.e., masked) by daytime light. This effect is mostly independent of daylength, but relies on how locomotor activity was measured since the modulatory effects reported above for wheel-running activity were not observed when general activity was recorded.

### Behavioral measures of meal anticipation

Most of the previous works that investigated FAA in Syrian hamsters used wheel-running activity to analyze this behavior [12, 14, 15]. The present study also measured general activity in food anticipatory behavior in this species, together with the modulatory effects of daytime light.

FAA can be measured by several means, such as wheel-running activity, general cage activity (via infrared motion sensors or i.p. implanted telemetry devices as here) or food-bin activity. Previous studies have already found differences according to the chosen behavioral assays. For instance, rats [9] with lesions in the paraventricular hypothalamic nuclei fail to express FAA in the wheel, while they display sizeable FAA at the food bin [27]. Our data clearly indicate that hungry hamsters are not prone to use the wheel before feeding time when they are exposed to regular light-dark cycles, either in long or short photoperiods. This observation is in keeping with previous findings in food-restricted Syrian hamsters [12, 14], unless they are challenged with calorie restriction [15]. It is noteworthy, however, that motion detectors of the present study indicate that food-restricted hamsters under full photoperiods exhibit FAA, suggesting that during the period preceding food access, the animals are active in their cage, probably close to the feeder, waiting for the time of food availability. Further discussion on the impact of measurement devices of meal anticipation can be found elsewhere [28].

### Masking effect of daytime light on meal anticipation

Light is a potent inhibitor of locomotor activity in nocturnal mammals, including the Syrian hamster [29]. Here this modulatory effect is particularly evident on the expression of hamster FAA in the wheel, confirming previous results in other nocturnal rodents, namely rats and mice [19]. Furthermore, this result is also in line with the apparent increase of FAA in the wheel when food-restricted hamsters are transferred from light-dark cycle to constant darkness (i.e., a condition that unmasks voluntary activity inhibited by daytime light [14]. This suppressing effect of light on FAA in the wheel does not mean, however, that the hamsters are hypoactive or resting during the pre-prandial because FAA in the same animals is detected by motion sensors. Therefore, the lack of FAA in the wheel may be a behavioral strategy of food-restricted hamsters to save energy. This explanation actually does not fit with the fact that lean calorie-restricted hamsters ultimately manifest FAA in the wheel under a full photoperiod [15]. Alternatively, the rewarding effect of wheel-running activity may be specifically inhibited by the combination of daytime light and restricted feeding. The neuronal pathways mediating this behavioral effect are not fully characterized yet and it is not clear whether the SCN may be involved. Bright light that inhibits wheel-running activity [29], is also known to activate (i.e., increase firing rate) SCN neuronal activity in nocturnal rodents [30, 31]. It is possible, however, that these photic cues impact on FAA behavior by bypassing the SCN itself [32], as already suggested for the direct effects of light on plasma glucose and corticosterone [33, 34].

### Photoperiodic changes in meal anticipation

Measure of daylength is critical for photoperiodic species, such as hamsters, whose reproductive and metabolic physiology drastically changes according to the seasons. Activity levels in the wheel are reduced in hamsters exposed to a very short photoperiod (i.e., 06-h light and 18-h dark cycle) [35, 36]. The smaller difference between the long and short photoperiods that were compared here may explain why we did not confirm this modulatory effect on daily levels of wheel-running activity. In the present context of food anticipation, variations in daylength may impact on brain activity via tonic effects of daytime light [37]. Even if lab mice are not photoperiodic in their reproduction or metabolism, they display longer FAA when exposed to long photoperiods compared to standard 12:12 light-dark cycle [32].

By contrast, FAA is increased in Siberian hamsters exposed to a short photoperiod, when they are sexually inactive. This effect seems not to be related to sex steroids because gonadectomy under long days does not mimic a short day-like anticipation [38]. In the present study, FAA in the wheel is not modified according to the photoperiod. Nevertheless, FAA in general activity is relatively increased when food restricted Syrian hamsters are exposed to long days as compared to short days. The photoperiodic modulation of meal anticipation thus appears to be opposite between Siberian and Syrian hamsters. Metabolic responses to photoperiod may account for this difference. Indeed, Syrian hamsters prepare for overwintering by increasing body lipids, while Siberian decrease body lipids and lean mass [20]. In Syrian hamsters, the SP-induced increased adiposity is usually associated with increased body weight [20], but not always [39](present study). It is tempting to speculate that body condition and related neuroendocrine factors could play some role in that seasonal modulation of FAA. Plasma leptin is secreted by adipocytes in relative proportion to adiposity [40]. Accordingly, levels of plasma leptin are high in Siberian hamsters exposed to SP and in Syrian hamsters exposed to LP [39]. In both Syrian and Siberian hamsters, high plasma leptin is concomitant with low FAA, but this occurs for different photoperiodic conditions in the two species [38]. In accordance with these negative correlations between plasma leptin and FAA amplitude, FAA in the wheel is also increased in *ob/ob* mice lacking leptin, while leptin treatment reduces the level of FAA [41]. FAA is also increased in another species with impaired leptin signaling, namely the Zucker rat (*Lepr* ^*fa*^ *)*) that has a missense mutation in the long form of leptin receptor [42]. In addition, assuming that brain structures regulating FAA are not leptin-resistant, diet-induced obesity in rats is another condition associating reduced FAA with increased adiposity and high levels of circulating leptin [43]. Together, these findings raise the possibility that leptin may negatively impact expression of FAA, including in Syrian hamsters.

### Molecular hypothalamic responses according to photoperiodic conditions

The brain mechanisms underlying FAA are not fully understood yet, although central clock mechanisms are likely involved [44]. It has been suggested that FAA is controlled by a food clock actually relying on a network of distributed brain oscillators interacting with peripheral cues that provide information on the metabolic status to the brain. Here we used c-Fos staining to evaluate hypothalamic activated areas during FAA in Syrian hamsters.

The ARC is a hypothalamic region deeply involved in the regulation of energy homeostasis. It contains 2 groups of specialized neurons containing either orexigenic (i.e., NPY and AGRP) or anorexigenic neuropeptides (i.e., POMC/CART (anorexigenic) [45]. In this work we saw that this region is highly activated in food-restricted hamsters, independently of the photoperiod. High c-Fos expression has already been described during FAA in the ARC of rats and mice [8, 11]. This c-Fos activation in food-restricted hamsters may be, in part, related to their body mass loss and/or metabolic changes due the fasting period since the last meal. It should be point out, however, that such increased c-Fos in the ARC can persist in mice refed with food *ad libitum*, suggesting its involvement in the circadian control of FAA [11].

The DMH is another structure implicated in energy metabolism. The involvement of DMH in FAA is still a subject of debate, this structure being considered as necessary [7] or not [46, 47]. Regarding c-Fos, we noticed an increase in the DMH of food-restricted hamsters only under SP, despite similar body mass loss in both SP and LP. This comparison suggests indirectly that the increased c-Fos in the DMH of SP hamsters is not solely a consequence of metabolic changes due to body mass loss and/or due to depletion of energy stores since the previous meal. It was unexpected to find a larger activation of c-Fos in RF hamsters in SP because food-restricted hamsters under SP express less FAA in general activity. Nevertheless, the c-Fos increase in SP is in accordance with the neuronal activation during FAA in the DMH of rats and mice [8, 11, 48–50]. As for the ARC, the persistence of DMH c-Fos in mice refed with food *ad libitum* supports its involvement in the circadian regulation of FAA [11].

The VMH is another hypothalamic region that plays an important role in the control of energy metabolism and food intake [45]. Activation of c-Fos expression was found during FAA in food-restricted hamsters, but only in LP. Again, this differential induction of c-Fos between SP and LP occurs despite similar body mass loss and similar fasting duration since the last meal, suggesting that metabolic cues are necessary, but not sufficient for activating the VMH. Previous reports did detect significant increased c-Fos in the VMH during meal anticipation [8, 11, 49, 51] or not [48]. Lesions in VMH attenuate or block FAA in rats but this effect is only transient, suggesting that the VMH is involved, to some extent, in the expression of FAA [52].

The SCN, site of the master clock, is known to be not critical for expression of FAA, as evidenced by lesion experiments [4]. Here we found no significant difference in c-Fos expression between food-restricted and control fed hamsters, in accordance with previous studies in rats [8, 48]. It should be noted, however, that other studies have reported either robust increase in rats [49] or significant decrease in rats and mice [6, 11].

A previous study raises the intriguing hypothesis that FAA in rats can only occur when the DMH inhibits neuronal activity of the SCN, via activation of GABA-RFRP neurons projecting from DMH to the SCN that would disinhibit expression of voluntary locomotor activity during daytime [6]. Studying the Syrian hamster provides an ideal model to test this hypothesis. Indeed, as photoperiodic species, Syrian hamsters exhibit drastic seasonal changes in gonadal activity, that are orchestrated by hypothalamic changes in kisspeptin and RFRP. More precisely, RFRP is highly expressed in a small group of neurons clustered at the boundary between ventral DMH and dorsal VMH in sexually active hamsters exposed to LP, while this neuropeptide is barely detectable in sexually quiescent hamsters transferred to SP [16, 53, 54]. Therefore, Syrian hamsters exposed to SP are equivalent to a knock-down of RFRP expression. Interestingly, the reduced FAA of general activity in food-restricted hamsters exposed to SP gives some support to the possibility that during SP, lower activity of (RFRP-containing) DMH neurons would no longer inhibit the SCN, thus impairing full expression of FAA during daytime. By contrast, the unaltered FAA in the wheel in SP, the lack of decreased c-Fos in the SCN in both SP and LP, and the lack of increased c-Fos in the DMH of food-restricted hamsters in LP are not in accordance with that hypothesis implicating a DMH-SCN pathway. Therefore, further studies using pharmacological tools (e.g., RFRP antagonists in LP) and neurochemical lesions are needed to investigate whether the DMH modulates the expression of FAA in hamsters and whether this effect involves the SCN.

## Conclusions

The main finding of this study is that FAA can be detected in hamsters that are food-restricted under both SP and LP, provided that locomotor activity is measured by general cage activity. Second, the presence of bright light during daytime is a major inhibitor of FAA expression in the wheel. Third, this study shows that the ARC, and to a less extent DMH and VMH are activated during FAA in hamsters, thus suggesting that these structures participate in the expression of FAA in that species. Further studies are needed in hamsters to clarify how daytime light negatively modulates FAA in the wheel and to better understand the neural network underlying circadian expression of meal anticipation.
